# Corretion to “Nonlinear Investigation of Fluorene-Benzothiadiazole Copolymers with Multiphoton Absorption and Highlights as Optical Limiters”

**DOI:** 10.1021/acsomega.5c06629

**Published:** 2025-07-30

**Authors:** Leandro H. Zucolotto Cocca, João V. P. Valverde, Elisa B. de Brito, Jilian Nei de Freitas, Maria de F. V. Marques, Cleber R. Mendonça, Leonardo De Boni

The correct ORCID for Prof. Maria de F. V. Marques is 0000-0002-8629-3419.

